# Portal venous gas: A benign finding in pyloric stenosis?

**DOI:** 10.1002/jpr3.70051

**Published:** 2025-07-07

**Authors:** Kelci Butler, Tiffany Patton

**Affiliations:** ^1^ Pediatric Hospital Medicine Advocate Children's Hospital Park Ridge Illinois USA; ^2^ Pediatric Gastroenterology Advocate Children's Hospital Oak Lawn Illinois USA

**Keywords:** intestinal disease, neonatal, pediatric gastroenterology

## Abstract

Pyloric stenosis is a condition of infancy characterized by hypertrophy of the pylorus, which can progress to significant narrowing and near‐obstruction of the gastric outlet. We describe a case of a patient with pyloric stenosis who was incidentally found to have portal venous gas on ultrasound. While the presence of portal venous gas can be an ominous finding, this case demonstrates that in the setting of pyloric stenosis, portal venous gas may be benign in nature. Additionally, we compile information from previous studies to further promote general recognition of this association and propose potential future standardization of care for these patients with an apparent benign finding.

## 1 INTRODUCTION

Infantile hypertrophic pyloric stenosis is a relatively common condition, with an incidence of 2–5 in 1000 live births. It most commonly develops in infancy between 3 and 5 weeks of age. It typically presents with nonbilious projectile emesis after feeding. Ultrasound is the gold standard for diagnosis to confirm the presence of a hypertrophic pyloric muscle, defined as pyloric wall thickness 3 mm or greater and pyloric channel length 15 mm or greater without passage of gastric contents through the thickened pyloric canal.^1^

The coexistence of hypertrophic pyloric stenosis and portal venous gas is rare, and its implications are poorly understood. There has only been one retrospective review of sonographic findings that found a prevalence of portal venous gas in approximately 1.8% of patients with pyloric stenosis.^2^ The pathophysiology of hepatic portal venous gas is not clearly defined. The etiology of portal venous gas can range from a benign, transient process to bowel necrosis.

We present a case of a 4‐week‐old female who presented with 2 weeks of progressive, forceful emesis and was diagnosed with pyloric stenosis. On ultrasound obtained to evaluate for pyloric stenosis, portal venous gas was incidentally identified.

## 2 CASE REPORT

**Figure 1:**
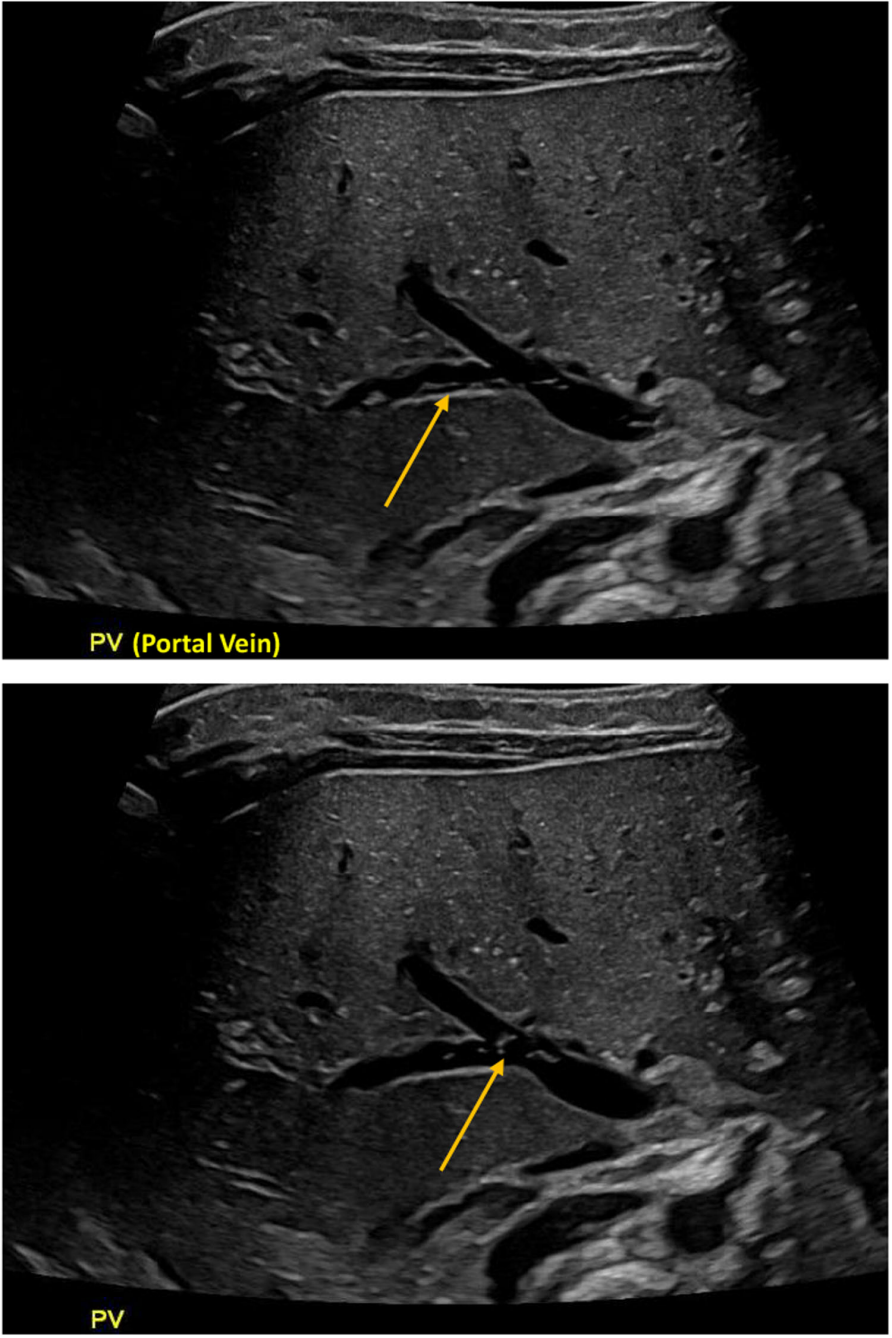
Two right upper quadrant ultrasound images showing multiple dynamic echogenic foci in the portal vein representing portal venous gas.

A 4‐week‐old female, born at 38 weeks' gestation, presented with 2 weeks of nonbilious emesis. The emesis was initially intermittent but became more frequent and projectile in nature. Pyloric ultrasound was consistent with pyloric stenosis with muscle wall thickness measuring 5 mm and pyloric channel length measuring 20 mm (normal wall thickness <3 mm; normal channel length < 15 mm). The ultrasound also visualized innumerable echogenicity throughout the included portion of liver parenchyma. This was further evaluated by right upper quadrant ultrasound, which showed multiple dynamic echogenic foci in the portal vein representing portal venous gas (Figure 1). There was bowel wall thickening without pneumatosis detected. A follow‐up 2‐view abdominal radiograph showed no gross pneumoperitoneum.

She was admitted to the pediatric intensive care unit for close monitoring due to concern for possible early/developing necrotizing enterocolitis while awaiting her scheduled pyloromyotomy. She was kept nil per os, had a nasogastric tube placed to suction, and was started on broad‐spectrum empiric antibiotics due to concern for necrotizing enterocolitis. Serial abdominal radiographs were obtained, none of which showed evidence of free air, pneumatosis intestinalis, or portal venous gas. She remained clinically well appearing with a benign abdominal exam. The following day, she underwent an uncomplicated pyloromyotomy. Gross inspection of the abdomen demonstrated healthy bowel. Postoperatively, antibiotics were discontinued, and her feeds were advanced as tolerated. A follow‐up right upper quadrant ultrasound was obtained and showed trace residual portal venous gas with an otherwise normal exam. She was subsequently discharged home on postoperative day 1. She was seen for follow‐up in the outpatient setting 3 weeks postoperatively and continued to do well, tolerate feeds without emesis, and gain appropriate weight.

## 3 DISCUSSION

In the patient described in our case, who was otherwise well appearing with stable vital signs and a benign physical exam, the portal venous gas that was identified was likely secondary to or associated with her diagnosis of pyloric stenosis. The exact pathophysiology of portal venous gas is not fully understood. Previous literature has supported the hypothesis of recurrent forceful emesis, as was seen in our patient, as an etiology of transient portal venous gas. This is explained by an escape of gas from increased intraluminal pressure of the bowels.^3^ The proposed pathophysiology for portal venous gas as seen in pyloric stenosis suggests a mechanical etiology, as opposed to infectious or ischemic, of gas escaping through intact mucosa into the portal venous system. In the setting of pyloric stenosis, the presence of portal venous gas should thus be transient with resolution following gastrointestinal decompression via pyloromyotomy.

In neonates, portal venous gas is highly concerning for necrotizing enterocolitis. Necrotizing enterocolitis is the most common intra‐abdominal surgical emergency in the neonatal population.^4^ It primarily affects preterm newborns but has also been reported in full‐term neonates. Patients with necrotizing enterocolitis may present with feeding intolerance, however, with progression of the disease process can present with abdominal distention, bloody stools, and lethargy. Ultimately, our patient was managed presumptively for necrotizing enterocolitis until an intraoperative evaluation could exclude this diagnosis.

Despite pyloric stenosis being a relatively common pediatric diagnosis, there is a paucity of literature describing the presence of concurrent portal venous gas and its implications. While there have been previously reported studies of portal venous gas associated with pyloric stenosis (Table 1), this is the first compilation of these studies to our knowledge. The management of these patients remains highly variable, with previous studies reporting a variety of evaluations ranging from no additional interventions to serial imaging and empiric antibiotics. Comparable to prior studies, surveillance imaging was obtained in our case, and there was ultimately resolution following pyloromyotomy. It is evident that there remains a gap in widespread recognition of this apparent benign association. Further, there remains an opportunity to standardize the management of patients with pyloric stenosis who have concomitant findings of portal venous gas. We would propose that it be appropriate for no further diagnostic or therapeutic interventions be performed in well‐appearing patients with pyloric stenosis who have incidental findings of portal venous gas. The finding of portal venous gas should not delay surgical intervention in patients with pyloric stenosis.

**Table 1:** Previously published studies of portal venous gas in the setting of pyloric stenosis.

Diagnosis associated with pyloric stenosis	Imaging obtained	Outcome described	Publication	Publication type
Portal venous gas	Abdominal ultrasound, duplex ultrasound of portal vein, abdominal plain films	Resolution following pyloromyotomy	Sorantin et al. [5]	Case report
Portal venous gas	Abdominal ultrasound, abdominal computed tomography (CT) scan	Benign course following pyloromyotomy	Sarti and Kennedy [6]	Case report
Gastric pneumatosis and portal venous gas	Abdominal ultrasound, abdominal plain films	Rapid resolution following pyloromyotomy	Bhargava and Parisi [7]	Case report
Portal venous gas and gastric pneumatosis	All with abdominal ultrasound (one case with serial ultrasounds) and abdominal plain films	Correlation recognized in 4 cases; outcomes not described	Cohen et al. [8]	Case series
Hepatic portal venous gas	Abdominal ultrasounds (serial), abdominal plain films, upper gastrointestinal series (serial), upper endoscopy	Resolution following pyloromyotomy	Daniel et al. [9]	Case report
Portal venous gas	Not described	No significant difference in outcome or length of hospital stay for patients with and without portal venous gas, the finding of portal venous gas appears benign	Kelly et al. [2]	Retrospective review
Hepatic portal venous gas	Abdominal ultrasound	Uncomplicated course following pyloromyotomy	Asemota et al. [10]	Case report

## 4 CONCLUSION

This report suggests that in a clinically well patient without other signs or symptoms of necrotizing enterocolitis, portal venous gas may be a benign finding associated with pyloric stenosis. For patients with a diagnosis of pyloric stenosis who are also found to have portal venous gas on ultrasound, there may not be a need for further investigation when there is otherwise low clinical suspicion for necrotizing enterocolitis. Further investigation could lead to potentially unnecessary radiation exposure and antibiotic overuse in the vulnerable neonatal population. At a system level, this could also lead to potentially unnecessary utilization of intensive care resources, prolonged length of stay, and consequential increase in healthcare costs.

## CONFLICT OF INTEREST STATEMENT

The authors declare no conflicts of interest.

## ETHICS STATEMENT

Written informed consent was obtained from the parent of the patient presented in this report, who understands and agrees to the publication of the case.
